# Establishment and Characterization of Human Germline Stem Cell Line with Unlimited Proliferation Potentials and no Tumor Formation

**DOI:** 10.1038/srep16922

**Published:** 2015-11-20

**Authors:** Jingmei Hou, Minghui Niu, Linhong Liu, Zijue Zhu, Xiaobo Wang, Min Sun, Qingqing Yuan, Shi Yang, Wenxian Zeng, Yang Liu, Zheng Li, Zuping He

**Affiliations:** 1 State Key Laboratory of Oncogenes and Related Genes, Renji-Med X Clinical Stem Cell Research Center, Ren Ji Hospital, School of Medicine, Shanghai Jiao Tong University, 160 Pujian Road, Shanghai 200127, China; 2 Department of Urology, Ren Ji Hospital, School of Medicine, Shanghai Jiao Tong University, Shanghai Institute of Andrology, 145 Shangdong Road, Shanghai 200001, China; 3 Shanghai Key Laboratory of Assisted Reproduction and Reproductive Genetics, Shanghai 200127, China; 4 Shanghai Key Laboratory of Reproductive Medicine, Shanghai 200025, China; 5 Northwest Agricultural & Forest University, Shaanxi, 712100, China; 6 Department of Andrology, Shanghai General Hospital, Shanghai Jiao Tong University, 100 Haining Road, Shanghai 200080, China

## Abstract

Spermatogonial stem cells (SSCs) have significant applications in both reproductive and regenerative medicine. However, primary human SSCs are very rare, and a human SSC line has not yet been available. In this study, we have for the first time reported a stable human SSC line by stably expressing human SV40 large T antigen. RT-PCR, immunocytochemistry, and Western blots revealed that this cell line was positive for a number of human spermatogonial and SSC hallmarks, including VASA, DAZL, MAGEA4, GFRA1, RET, UCHL1, GPR125, PLZF and THY1, suggesting that these cells are human SSCs phenotypically. Proliferation analysis showed that the cell line could be expanded with significant increases of cells for 1.5 years, and high levels of PCNA, UCHL1 and SV40 were maintained for long-term culture. Transplantation assay indicated that human SSC line was able to colonize and proliferate *in vivo* in the recipient mice. Neither Y chromosome microdeletions of numerous genes nor tumor formation was observed in human SSC line although there was abnormal karyotype in this cell line. Collectively, we have established a human SSC line with unlimited proliferation potentials and no tumorgenesis, which could provide an abundant source of human SSCs for their mechanistic studies and translational medicine.

Spermatogonial stem cells (SSCs) are a subpopulation of type A spermatogonia. Studies on SSCs are of unusual significance in view of their unique characteristics^1^. Firstly, SSCs are the only adult stem cells that transmit genetic information to subsequent generations, and thus they represent an invaluable resource for experimental modification of the mammalian genome^2^. Secondly, SSCs self-renew throughout mammalian whole life and they differentiate into spermatocytes and mature spermatozoa, and therefore they can be utilized as an excellent model to uncover the molecular mechanisms underlying the renewal versus differentiation of stem cells. Thirdly, it might be feasible to use SSC transplantation to restore fertility in cancer patients after chemotherapy and/or irradiation therapy^3^. Male infertility has become a major health and social concern worldwide, due to environmental factors, inflammation, and anti-tumor therapy^4^. It has been reported that infertility affects around 15% of couples and male factors account for 50%^5^. Azoospermia has been found in 1% of the general populations and it comprises 10–15% male infertility^6^. We have recently shown that human SSCs can be induced to differentiate into haploid spermatids with fertilization and developmental capacity^7^, reflecting that they can provide mature and functional male gametes for azoospermic patients with SSCs. Lastly and more importantly, a number of studies have demonstrated that SSCs can acquire pluripotency to become embryonic stem (ES)-like cells that are able to differentiate into all cell lineages of three germ cell layers^8^,^9^,^10^,^11^,^12^,^13^. Strikingly, numerous studies by peer and us have recently demonstrated that SSCs are able to directly transdifferentiate into the cells of other lineages both *in vivo* and *in vitro*^14^,^15^,^16^. Therefore, SSCs have great applications on regenerative medicine without the associated ethical issues and immune rejection.

Notably, very little information is available on the biology of human SSCs due to the following factors: i) it is rather difficult to obtain human testicular tissues; ii) the number of human SSCs is very few. It has been reported that SSCs represent only 0.03% of all germ cells in rodent testis^17^, and there is only 1 or 2 human SSCs in each cross section of the seminiferous tubules^18^; iii) it is rather hard to expand human SSCs *in vitro* and a long-term culture system of human SSCs has not yet been established. The limited life-span and rare number of human SSCs represent a serious problem for understanding molecular mechanisms of human spermatogenesis; and iv) there is not yet a human SSC line to obtain sufficient cells for their usage from the bench to bed side. Spermatogonial cell line and SSC line have been set up using plasmids over-expressing telomerase or SV40 large T antigen in rodents^19^,^20^. Nevertheless, a human SSC line is currently unavailable. Notably, there are distinct identity and cell types for rodent and human SSCs, since the A_s_ spermatogonia are the actual stem cells for rodents, while the A_dark_ and A_pale_ spermatogonia are generally regarded as human SSCs. Moreover, the phenotypic characteristics between rodent and human SSCs are different. As examples, OCT-4 (also known as POU5F1) is a hallmark for mouse SSCs, whereas it is absent in human SSCs^18^. Therefore, the mechanisms regulating fate decisions of human and rodent SSCs are distinct. Here we have for the first time reported a human SSC line by stably introducing SV40 large T antigen through lentivirus infection. Cellular, molecular, and functional assays *in vitro* and *in vivo* revealed that this cell line was human SSCs without Y chromosome microdeletions of numerous genes or tumor formation and it could be expanded with significant increases of cell number *in vitro* for over one and half years and colonized *in vivo* in the recipient mice. Significantly, our ability of establishing human SSC line could offer an unlimited cell source of human SSCs for their basic studies and great applications in regenerative and reproductive medicine.

## Results

### Immortalization of Human Male Germline Stem Cells

**Figure 1:**
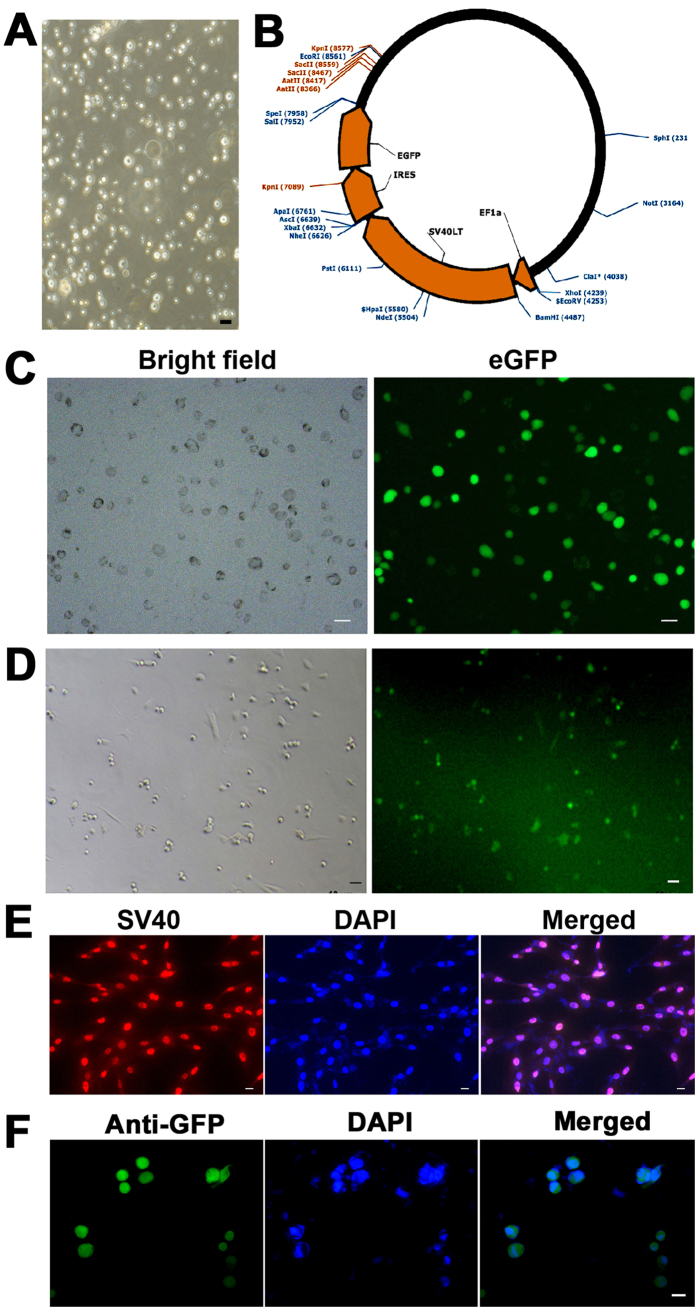
Immortalization of human male germline stem cells. (**A**) Human male germ cells were isolated from testicular tissues of OA patients by two enzymatic digestions and differential plating. (**B**) The diagram showed the structure of lentivirus namely Lenti-EF1α-SV40LargeT-IRES-eGFP. (**C**) The bright filed (left panel) and eGFP expression (right panel) of human male germ cells at 24 hours after transfection of Lenti-EF1α-SV40LargeT-IRES-eGFP. (**D**) The bright filed (left panel) and eGFP expression (right panel) of human SSC line from the immortalized human male germ cells after MACS using an antibody against GPR125. (**E,F**) Immunochemistry displayed the expression of SV40 protein (**E**) and GFP (**F**) in the immortalized human SSCs. Scale bar in A = 30 μm; scale bars in (**C,D**) = 20 μm; scale bars in E and F = 10 μm.

Human male germ cells were separated from testicular tissues of obstructive azoospermic (OA) patients using two enzymatic digestions and followed by differential plating (Fig. 1A), and they were transfected with lentivirus called Lenti-EF1α-SV40LargeT-IRES-eGFP (Fig. 1B) by polybrene. The expression of SV40 large T antigen was driven by the promoter of EF1α, and eGFP was utilized as a reporter gene. After 24 hours of transfection, eGFP expression was observed in human male germ cells under a fluorescence microscope (Fig. 1C). The immortalized human SSCs (Fig. 1D, left panel) were isolated and purified by MACS using an antibody against GPR125, and their stable eGFP expression was seen (Fig. 1D, right panel). In morphology, the immortalized human SSCs assumed oval shapes with similar diameters of 10 μm under a phase-contrast microscopy.

**Figure 2:**
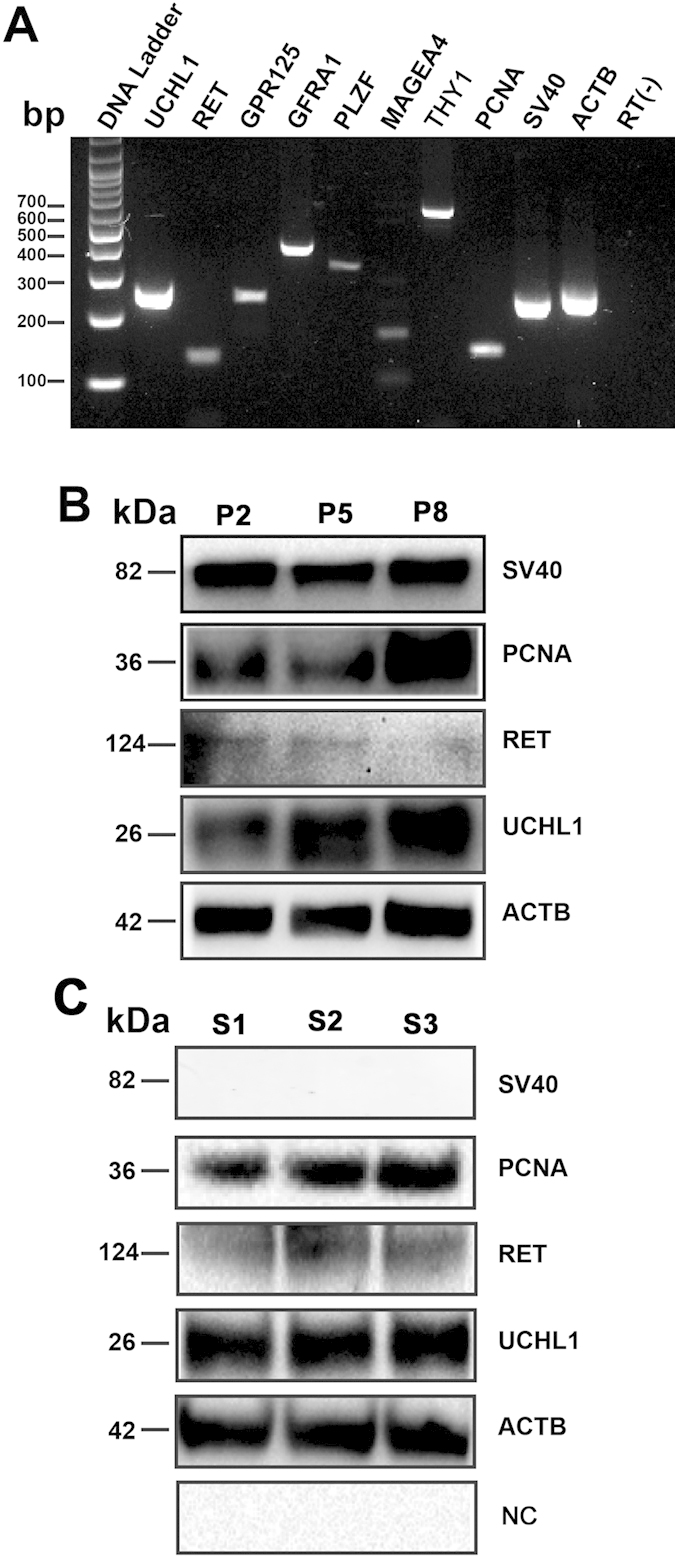
Phenotypic characteristics of the immortalized human male germline stem cells. (**A**) RT-PCR showed the expression of *UCHL1, RET, GPR125, GFRA1, PLZF, MAGEA4, THY1, PCNA* and *SV40* in the immortalized human male germline stem cells. *ACTB* was used as a loading control of total RNA, whereas RNA without RT (RT-) but with PCR served as a negative control. (**B,C**) Western blots revealed the expression of SV40, PCNA, UCHL1, and RET in the human SSC line (**B**) at different passages and primary human SSCs (**C**). ACTB was utilized as a loading control of loading proteins, while replacement of primary antibodies with PBS served as negative controls (NC). Notes: passage 2 (P2); passage 5 (P5); passage 8 (P8); S1, S2, and S3 indicated 3 independent experiments of primary human SSCs.

Moreover, immunnostaining revealed that SV40 protein (Fig. 1E) and GFP (Fig. 1F) was stably expressed in the immortalized human SSCs. RT-PCR and Western blots further demonstrated that *SV40 Large T antigen gene* (*SV40)* (Fig. 2A) and SV40 protein (Fig. 2B) were also detected in these cells. In contrast, the protein of SV40 was undetected in primary human SSCs (Fig. 2C), thus verifying specific expression of SV40 protein in the immortalized human SSCs.

### Phenotypic Identification of the Immortalized Human Male Germline Stem Cells

It is essential to check whether the immortalized human male germline stem cells were human SSCs in phenotype. To this end, we detected a series of markers for human SSCs using RT-PCR, Western blots, and immunocytochemical staining. RT-PCR revealed that the transcripts of *UCHL1*, *RET*, *GPR125*, *GFRA1*, *PLZF*, *MAGEA4* and *THY1* were detected in the immortalized human SSCs (Fig. 2A). Western blots showed that the proteins of UCHL1 and RET were expressed in human SSC line at passage 2 (P2), P5, and P8 (Fig. 2B). In parallel, the protein of UCHL1 and RET was detected in primary human SSCs (Fig. 2C), whereas replacement of the primary antibodies with PBS and no blots was observed, thus verifying specific expression of UCHL1 and RET in the immortalized human SSCs.

**Figure 3:**
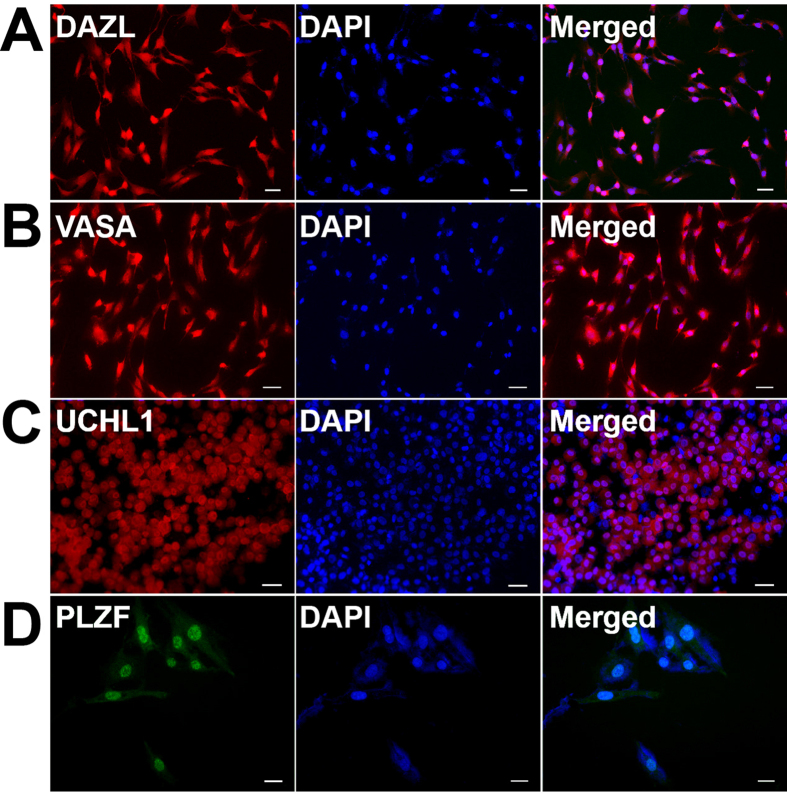
Biochemical features of the immortalized human male germline stem cells. (**A–D**) Immunocytochemistry demonstrated the expression of DAZL (**A**), VASA (**B**), UCHL1 (**C**), and PLZF (**D**) in human SSC line. Scale bars in (**A–D**) = 20 μm.

**Figure 4:**
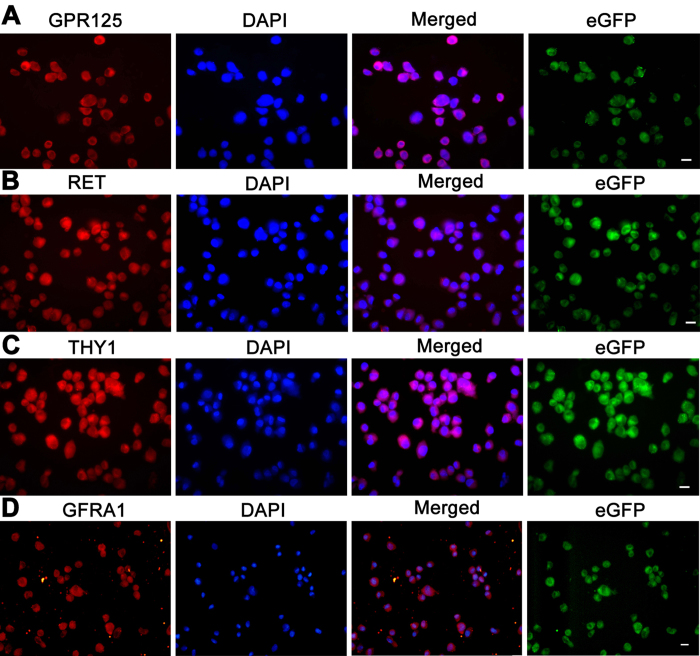
Phenotypic features of the immortalized human male germline stem cells. (**A–D**) Immunocytochemistry showed the expression of GPR125 (**A**), RET (**B**), THY1 (**C**), and GFRA1 (**D**), in human SSC line. eGFP expression of these cells was shown in the right panels. Scale bars in (**A–D**) = 10 μm.

Furthermore, immunocytochemistry displayed that DAZL (Fig. 3A, cultured cells), VASA (Fig. 3B, cultured cells), UCHL1 (Fig. 3C, the cells using cytospin), PLZF (Fig. 3D, cultured cells), GPR125 (Fig. 4A), RET (Fig. 4B), THY1 (also known as CD90) (Fig. 4C), and GFRA1 (Fig. 4D), markers for human germ cells and SSCs, were expressed in the immortalized human male germline stem cells. Considered together, these results suggest that the immortalized human male germline stem cells were human SSCs phenotypically.

### Proliferation Potentials of the Immortalized Human Male Germline Stem Cells

**Figure 5:**
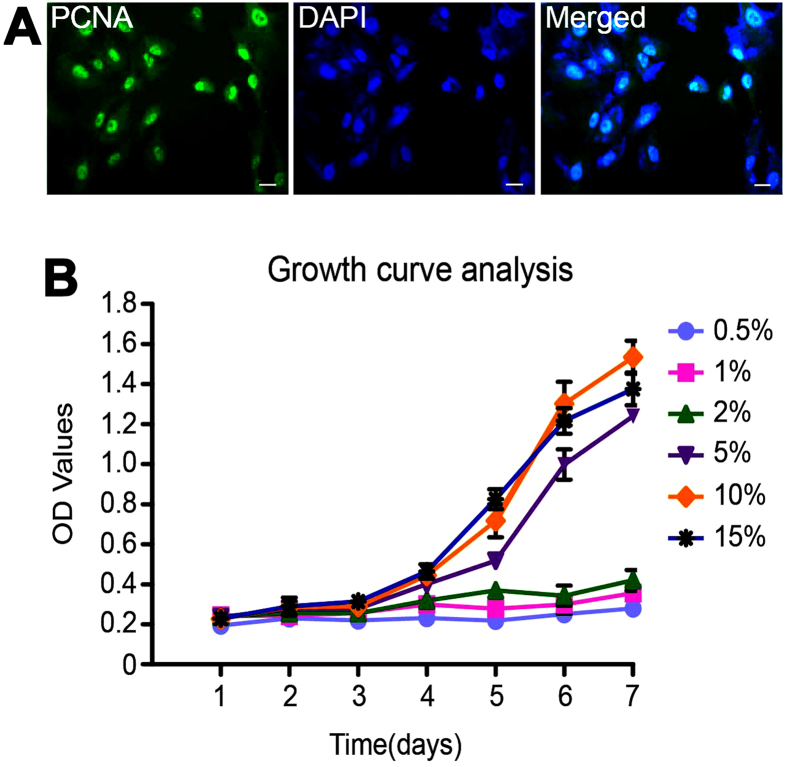
Proliferation capacity of the immortalized human male germline stem cells *in vitro*. (**A**) Immunocytochemistry revealed the expression of PCNA in human SSC line. Scale bar in (**A**) = 10 μm. (**B**) CCK-8 assays were performed to compare the effect of various kinds of FBS from 0.5% to 15% on the growth of human SSC line.

We next evaluated the proliferation potentials of the immortalized human male germline stem cells using various approaches. RT-PCR revealed that *PCNA* transcript was present in the immortalized human male germline stem cells (Fig. 2A), and Western blots displayed an increase of PCNA protein in human SSC line from P2 to P8 (Fig. 2B), indicating a higher proliferation capacity of these cells after passages. Immunocytochemistry further showed that the immortalized human male germline stem cell was positive for PCNA (Fig. 5A).

We also determined the effect of various concentrations of fetal bovine serum (FBS) in the proliferation ability of the immortalized human male germline stem cells. The immortalized cells appeared an obviously stronger proliferation than primary human SSCs, with a doubling time of cell number approximately 2–3 days. CCK8 assays showed that the proliferation of the immortalized human male germline stem cells was dependent on the concentrations of FBS (Fig. 5B). There was no obvious change of cell proliferation in the medium supplemented with 0.5%, 1%, and 2% FBS, and notably, 10% FBS was the best for the growth and expansion of this cell line (Fig. 5B). To date, this human SSC line had been cultured for 18 months in the medium DMEM/F12 with 10% FBS for more than 30 passages. During the whole period of culture and different passages, this cell line didn’t show morphological change or contact inhibition.

### The Immortalized Human Male Germline Stem Cells Colonized and Proliferated *in vivo* in the Recipient Mice

**Figure 6:**
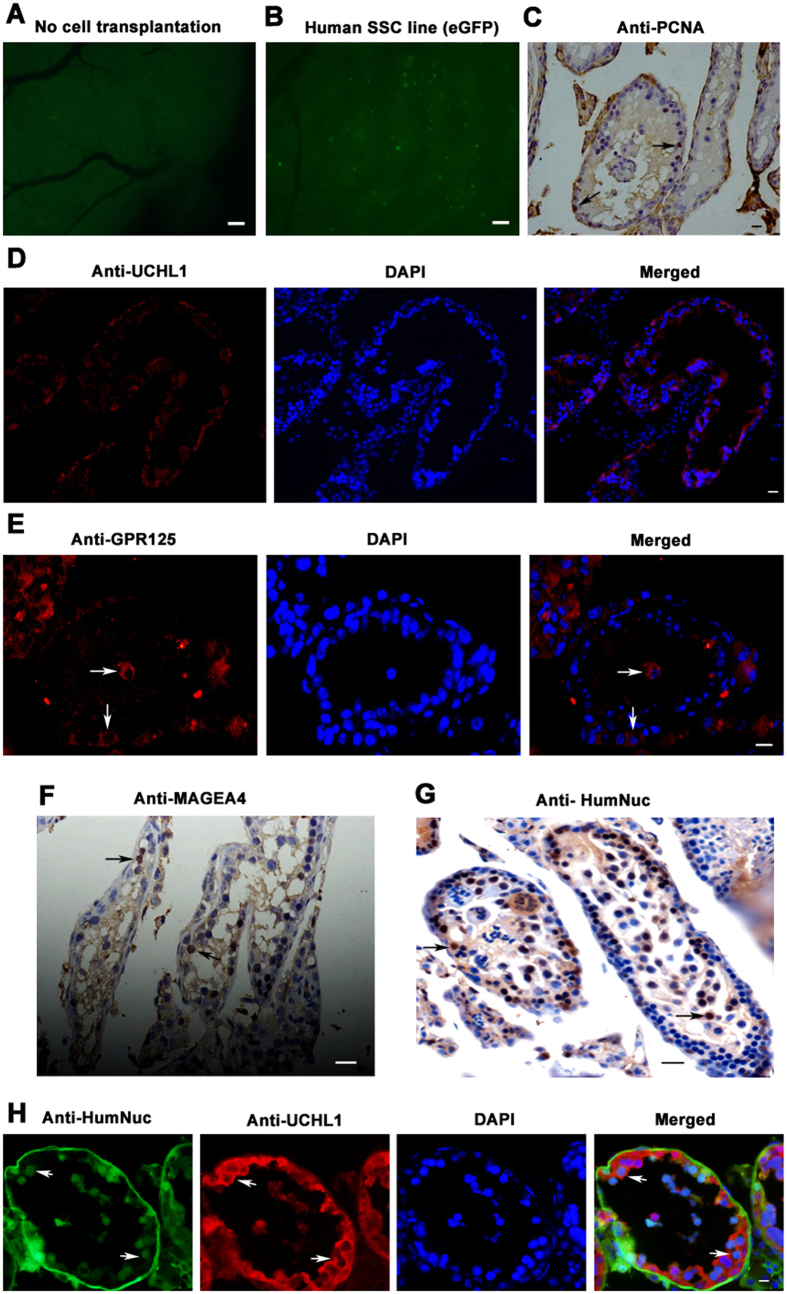
Functional assays of the immortalized human male germline stem cells *in vivo*. (**A,B**) The expression of eGFP in the control seminiferous tubules of recipient mice without cell transplantation (**A**) and with transplantation of the immortalized human male germline stem cells (**B**). Scale bars in (**A,B**) = 200 μm. (**C–H**) Immunohistochemisty illustrated the expression of PCNA (**C**), UCHL1 (**D**), GPR125 (**E**), MAGEA4 (**F**), and HumNuc (**G**), as well as co-expression of UCHL1 and HumNuc (**H**) in the seminiferous tubules of recipient mice with transplantation of the immortalized human male germline stem cells. Scale bars in (**C–E**) = 20 μm; scale bar in (**F,G**) = 30 μm; scale bar in (**H**) = 10 μm.

We further determined whether the immortalized human male germline stem cells could colonize and proliferate *in vivo* in the recipient mice. In total, 1.5 × 10^5^ cells were transplanted into each testis of recipient mice pre-treated with busulfan to eliminate male germ cells. Two months after transplantation, cell colonies expressing eGFP (Fig. 6B) were observed within the seminiferous tubules of recipient mice with transplantation of the immortalized human male germline stem cells, whereas no eGFP expression was seen in control seminiferous tubules of recipient mice without cell transplantation (Fig. 6A). Immunohistochemisty revealed that UCHL1 was expressed in the cells along the basement membrane of seminiferous tubules of recipient mice with transplantation of the immortalized human male germline stem cells (Fig. 6D). These data reflect that the immortalized human male germline stem cells could settle down and colonize in the seminiferous tubules of recipient mice. Moreover, immunohistochemistry demonstrated that PCNA, a marker for cell proliferation, was detected in the cells within the seminiferous tubules of recipient mice with transplantation of the immortalized human male germline stem cells (Fig. 6C). These results implicate that the immortalized human male germline stem cells could proliferate *in vivo* in recipient mice. Furthermore, immunohistochemistry revealed that GPR125 (Fig. 6E) and MAGEA4 (Fig. 6F), hallmarks for human SSCs and human spermatogonia, were present in the recipient mice. We also utilized an antibody to human nuclear antigen (HumNuc) that specifically recognized human antigens rather than mouse proteins. As shown in Fig. 6G, HumNuc was positive for the cells within the seminiferous tubules of recipient cells, reflecting the identity of the cells was originated from the immortalized human male germline stem cells. Double immunostaining further displayed that HumNuc and UCHL1 were co-expressed in the cells within seminiferous tubules of recipient mice (Fig. 6H). Collectively, these results clearly implicate that the immortalized human male germline stem cells could survive, proliferate and colonize in the recipient mice.

### The Immortalized Human Male Germline Stem Cells Showed Mutation in Karyotype but Excluded Y Chromosome Microdeletions of Numerous Genes

**Figure 7:**
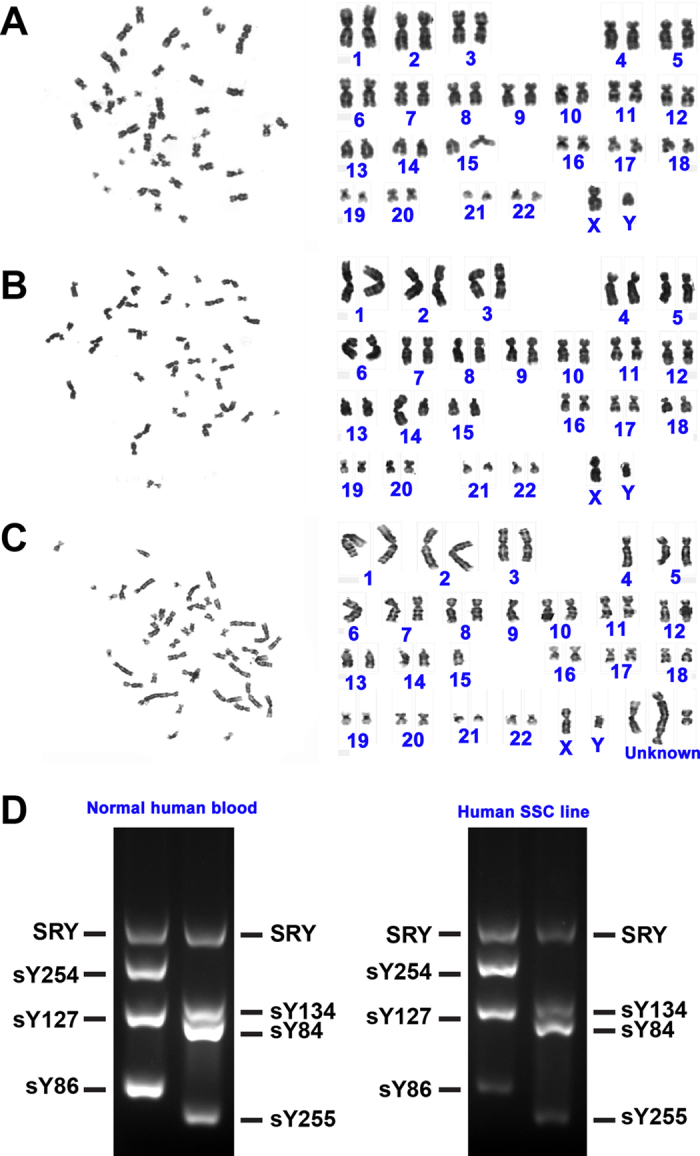
Safety evaluation of the immortalized human male germline stem cells. (**A–C**) Cytogenetic assay revealed normal (**A**) and abnormal karyotype (**B,C**) in the immortalized human male germline stem cells. (**D**) Multiplex PCR showed a number of Y chromosome genes, including SRY, sY254, sY127, sY86, sY134, sY84 and sY255, in the immortalized human male germline stem cells. The expression of these genes in normal human blood served as a positive control.

We also assessed whether transduction of human SSC cells with SV40 large T antigen could induce chromosomal modifications. Cytogenetic analysis showed that over 70% of the immortalized human male germline stem cells assumed a normal karyotype with 23 pairs of chromosomes (Fig. 7A), while around 30% of these cells had abnormal karyotype with unbalanced translocation or chromosomal numerical aberrations (Fig. 7B,C).

Multiplex PCR was utilized to estimate whether this cell line had gene microdeletions in Y chromosome. We detected numerous Y chromosome genes in the human immortalized cell line and normal human blood cells served as a positive control. As shown in Fig. 7D, a number of Y chromosome genes, including SRY, sY254, sY127, sY86, sY134, sY84 and sY255, were detected in the immortalized human male germline stem cells, suggesting that this cell line excludes Y chromosome microdeletions of these genes.

### The Immortalized Human Male Germline Stem Cells Didn’t Form Tumors in Xenografting Mice

**Figure 8:**
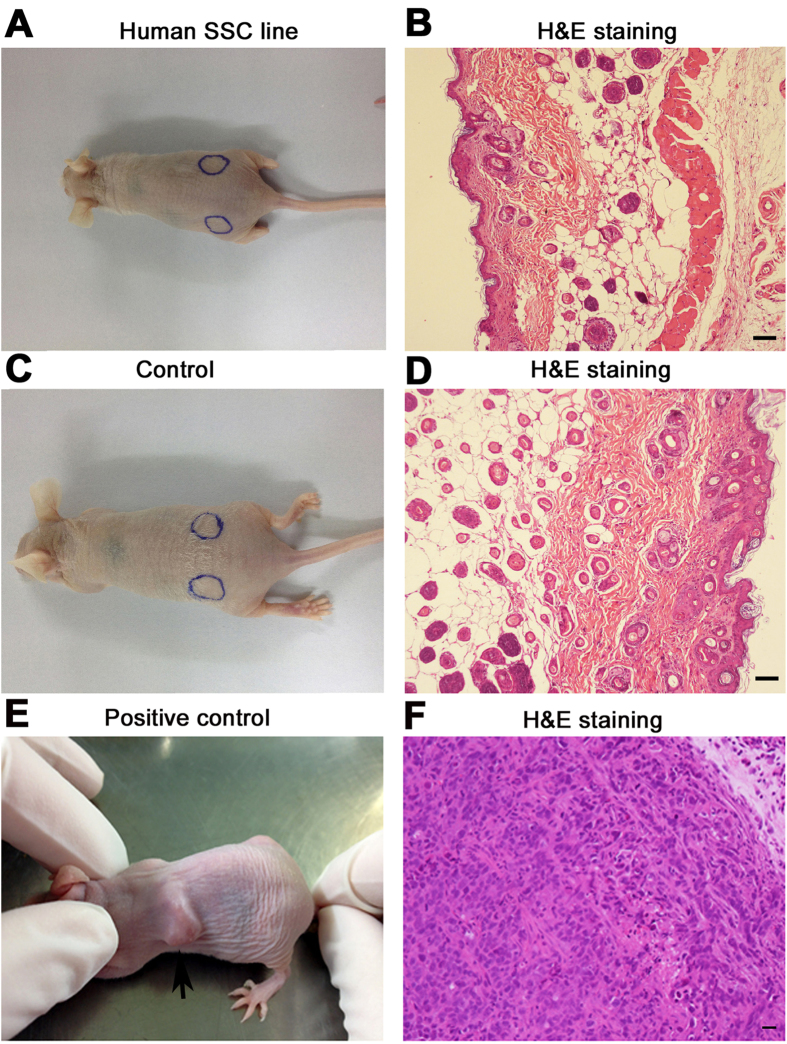
Tumor formation of the immortalized human male germline stem cells by nude mouse xenografting assays. (**A–F**) Digital camera and H&E staining revealed no tumor formation in recipient mice with transplantation of the immortalized human male germline stem cells (**A,B**) or without cell transgrafting (**C,D**) or with human prostate cancer cells DU145 (**E**,**F**). Arrow showed the tumors. Scale bars in (**A–D**) = 10 μm, and scale bars in (**E,F**) = 30 μm.

We finally asked whether the immortalized human male germline stem cells could form tumors using a nude mouse xenograft model. After 8 weeks of cell transplantation, no tumor formation was found at each skin site of recipient mice (Fig. 8A). H&E staining showed that there was regular and normal structure in the skin sites of transplantation (Fig. 8B). In parallel, no tumor formation was seen in the recipient mice without cell transplantation (Fig. 8C,D), whereas tumors were observed in the recipient mice with transplantation of prostate cancer cells DU145 (Fig. 8E,F).

## Discussion

Male germline stem cells could have significant applications in reproductive and regenerative medicine. Although much progress has been made on genetic and epigenetic regulation of SSCs in rodents^1^,^21^,^22^,^23^,^24^,^25^,^26^, it is hardly known about the molecular mechanisms underlying the process of human spermatogenesis. One of the urgent problems to be solved when working with human SSCs is the limited number of these cells. Additionally, human primary SSCs proliferate slowly *in vitro* and a long-term culture system to expand human SSCs has not yet been available. Therefore, it is essential to immortalize human SSCs to obtain sufficient cells for their basic research and clinic applications. There are spermatogonial line and SSC line in rodents^19^,^20^. Nevertheless, no human SSC line has been reported. In this study, we have for the first time established a stable human SSC line using lentivirus over-expressing SV40 large T antigen. It is feasible to immortalize rodent cells by transfection with the SV40 large T antigen^19^. Here we demonstrated that SV40 was stably expressed at both transcriptional and translational levels in human SSC line, as shown by RT-PCR, Western blots, and immunocytochemistry. Morphologically, the immortalized cells were oval in shape and their sizes were similar to the freshly isolated human SSCs, while these cells could attach more easily to the culture dishes or plates for growing and proliferating.

Notably, this human SSC line possess phenotypic characteristics of human primary SSCs, as evidenced by our observations that a number of markers for germ cells and SSCs, including DAZL, VASA, UCHL1, PLZF, GFRA1, RET, GPR125, and THY1, were detected in these cells. To determine that the immortalized cell line was of germ cell origin, we examined the expression of markers DAZL and VASA by immunocytochemistry. DAZL is an autosomally located gene in human testis and it is present in the nuclei and cytoplasm in fetal gonocytes^27^,^28^, while VASA is a member of the DEAD-box family of RNA helicases and it is only expressed in germ cell lineages^29^. We showed that proteins of DAZL and VASA were expressed in the immortalized cell line, suggesting that these cells were originated from germ cells. Furthermore, we investigated a series of markers specific for spermatogonia and SSCs to determine if this cell line was derived from SSCs. UCHL1 is positive for SSCs and other undifferentiated spermatogonia, and it is expressed in GPR125-positive spermatogonia isolated from adult human testes^18^. Human primary SSCs are positive for some markers of spermatogonia identified in other species, e.g., GFRA1, RET, PLZF, and MAGEA4. GFRA1 and RET are co-receptors for GDNF and they have been regarded as markers for SSCs^30^,^31^,^32^. PLZF has been characterized as a hallmark for mouse and adult monkey SSCs^25^,^33^. In recent studies, it has also been reported that PLZF is defined to human SSCs^18^,^34^. MAGEA4, a member of the cancer-testis antigen family, has also been shown to be expressed in human spermatogonia^18^, and THY1 (CD90) has been considered a surface marker for mouse and human SSCs^18^,^26^. It is worth noting that the transcripts and proteins of these markers mentioned above for germ cells as well as spermatogonia and SSCs were clearly detected in human SSC line, implicating that this cell line assumes biochemical features of human SSCs.

Human primary SSCs are difficult to survive for a long period of culture with a limited proliferation potential *in vitro*. Significantly, our human SSC cell line was able to be cultured *in vitro* for over one and half years without morphological change and their growth rate was remarkably higher than that of freshly isolated human SSCs. Furthermore, the expression of PCNA, a specific marker for cellular proliferation, was increasingly enhanced in human SSC line from passage 2 to passage 8. Interestingly, we demonstrate that this cell line can be cultured for a long period and expanded with a significant increase of cell number in DMEM/F12 supplemented only with 10% FBS but without the addition of any growth factor, which could provide abundant human SSCs for their mechanism studies and applications in clinic.

Spermatogonial transplantation is the unique functional assay *in vivo* for SSCs, which was first described in 1994 using mice as a model^35^,^36^. SSC is the only cell that can migrate to the basement membrane of seminiferous tubules and form clusters on the basement of seminiferous tubules^37^. This golden standard method has been successfully used in mice and rats, and it has recently been shown in monkey by homotransplantations^38^. For xenotransplantation of primate or human SSCs, it remains unclear whether these cells are able to survive for a long time in seminiferous tubules of recipient rodents. In the present study, the immortalized human SSCs could settle down and colonize in the seminiferous tubules of recipient mouse testes for more than two months, and notably, they were able to migrate to the basement membrane of seminiferous tubules and proliferate. The expression of UCHL1, GPR125, MAGEA4, HumNuc, and PCNA further verified their human spermatogonial stem cell properties. Nevertheless, no further differentiation of the human SSCs cell line could be detected due to the microenvironment of mouse testes, which is consistent with previous study showing that xenografting of primary human SSCs into the testes of recipient mice resulted in cell turnover but without differentiating into spermatocytes or spermatids^39^. Thus, it seems that both our human SSC line and primary human SSCs prefer self-renewal above differentiation under rodent testicular niche. These results manifest that our cell line possesses primary human SSC cell characteristics *in vivo*, and significantly, this cell line would be very useful to examine the molecular mechanisms underlying the proliferation and survival of human SSCs.

To further evaluate the safety of human SSC line, we checked karyotype, Y chromosome microdeletions, and tumor formation of these cells. Karyotyping demonstrates chromosomal changes involving in chromosomal number and detects chromosomal aberrations in the immortalization process. The majority of human SSC cell line had normal chromosomal characteristics, which is in agreement with a number of studies showing that, in the progress of cell line immortalization, chromosomal numerical aberrations and unbalanced translocations appeared. As examples, immortalized human adipose-derived stromal cells transduced with the human telomerase reverse transcriptase (hTERT) gene in combination with either SV40 or HPV E6/E7 genes shows some chromosomal aberrations^40^, and human hematopoietic stem cell line immortalized by successive co-expression of HPV16 E6/E7 and hTERT also exhibits structural or numerical chromosomal changes^41^. Notably, neither Y chromosome microdeletions of numerous genes nor tumor formation was seen in our human SSC line, highlighting significant applications of these cells in reproductive and regenerative medicine.

In conclusion, we have for the first time established a stable human SSC line that assumes the morphological, phenotypic, and functional attributes of human primary SSCs. This cell line could colonize and proliferate in recipient mouse testes after xenotransplantation. Significantly, this cell line excludes Y chromosome microdeletions of a number of genes and tumor formation and could be expanded with a remarkable increase of cells, which could offer cells for uncovering the molecular mechanisms underlying human SSC proliferation and survival and early stages of human spermatogenesis as well as the applications of human SSCs in treating male infertility and other human diseases.

## Methods

### Procurement of Testicular Biopsies from Obstructive Azoospermic (OA) Patients

Testicular biopsies were obtained from OA patients who underwent microdissection and testicular sperm extraction at Ren Ji Hospital affiliated to Shanghai Jiao Tong University School of Medicine. All OA patients had normal spermatogenesis, and they were caused by inflammation and vasoligation but not by congenital absence of the vas deferens or other diseases including cancer.

This study was approved by the Institutional Ethical Review Committee of Ren Ji Hospital (license number of ethics statement: 2012-01), Shanghai Jiao Tong University School of Medicine, and an informed consent of testicular tissues for research only was obtained from all subjects. All experiments were performed in accordance with relevant guidelines and regulation of the Institutional Ethical Review Committee of Ren Ji Hospital.

### Isolation of Human Male Germline Stem Cells

Testicular tissues from OA patients were washed three time in Dulbecco modified Eagle medium (DMEM) (Gibco) with antibiotics containing penicillin and streptomycin (Gibco) to remove potential contamination of Leydig cells and myoid cells. Human seminiferous tubules were isolated from testicular tissues by the first enzymatic digestion utilizing 2 mg/ml collagenase IV (Gibco) and 1 μg/μl DNase I (Gibco) in 34 °C water bath for 15 min according to the procedures described previously^18^,^42^,^43^,^44^. Male germ cells were further isolated from seminiferous tubules using a second enzymatic digestion with 4 mg/ml collagenase IV, 2.5 mg/ml hyaluronidase (Sigma), 2 mg/ml trypsin (Sigma), and 1 μg/μl DNase I and followed by differential plating^18^. For differential plating, cell mixture suspension was seeded into tissue culture dishes in DMEM/F12 supplemented with 10% FBS (Gibco) and incubated at 34 °C in 5% CO_2_ for 3 hours. When Sertoli cells attached to the dishes, human male germ cells remained in suspension and they were collected by centrifuging at 1000 rpm for 5 min.

### Immortalization of Human Male germline Stem Cells

The lentivirus, namely Lenti-EF1α-SV40LargeT-IRES-eGFP, was purchased from Sidansai Biotechnology CO., LTD (Shanghai, China). Human male germ cells were transfected with Lenti-EF1α-SV40LargeT-IRES-eGFP according to the manufacturer’s instruction. Briefly, human male germ cells were seeded at a density of 2 × 10^5^ cells/well in 24-well plates, and they were cultured with DMEM/F12 supplemented with 10% FBS for 3 hours. Half medium was removed and replenished with fresh DMEM/F12 medium consisting of 10^8^ TU/ml Lenti-EF1α-SV40LargeT-IRES-eGFP and 10 μg/ml polybrene, and the cells were incubated at 34 °C in 5% CO_2_ overnight. After 24 hours of culture, the medium was changed with fresh DMEM/F12 and 10% FBS and the expression of eGFP was detected under a fluorescence microscope (Nikon Eclipse Ti-S, Nikon Corporation, Tokyo, Japan). The transfected cells were cultured and expanded in DMEM/F12 medium supplemented with 10% FBS and 1% antibiotic containing penicillin and streptomycin (Gibco).

We have recently identified GPR125 as a hallmark for human SSCs^18^. Human SSCs were isolated and purified from the immortalized human male germ cells using magnetic-activated cell sorting (MACS) with an antibody against GPR125 (Abcam) pursuant to the method as previously described^18^,^45^. The immortalized human SSCs were cultured with DMEM/F12 supplemented with 10%FBS.

### RNA Extraction and Reverse Transcription-polymerase Chain Reaction (RT-PCR)

Total RNA was extracted from the immortalized human male germline stem cells using Trizol (Invitrogen). The cDNA was synthesized using the First Strand cDNA Synthesis Kit (Thermo Scientific) and PCR was performed according to the protocol as described previously^46^. The forward and reverse primers and PCR products of the chosen genes, including *UCHL1*, *RET*, *GPR125*, *GFRA1*, *PLZF*, *MAGEA4*, *THY1*, *PCNA*, *SV40*, and *ACTB*, were designed and listed in Table 1. The PCR reactions started at 94 °C for 5 min and were performed using the follow conditions: 30 sec at 95 °C, annealing temperature (49–58 °C) for 30 sec, 72 °C for 30 sec, 35cycles, and extended at 72 °C for 10 min. PCR products were separated by electrophoresis on 2.0% agarose, and the gels were exposed to chemiluminescence (Chemi-Doc XRS, Bio-Rad, Hercules, CA). Total RNA without RT (RT-) but with PCR using *GAPDH* primers served as a negative control.

### Immunocytochemistry of the Immortalized Human Male Germline Stem Cells

The phenotypic characteristics of the immortalized human male germline stem cells were identified by immunocytochemistry according to the procedure described previously^46^. The cultured cells or the cells using cytospin were fixed with 4% paraformaldehyde (PFA) and permeabilized in 0.4% triton-X 100 (Sigma-Aldrich) for 15 min at room temperature and followed by three washes in phosphate-buffered saline (PBS) for 5 min each. After blocking in 1% bovine serum albumin for 1 hour, the cells were incubated with primary antibodies, including SV40 (Santa Cruz), GFP (Abcam), DAZL (Abcam), VASA (Abcam), RET (Santa Cruz), UCHL1 (AbD Serotec), PLZF (Abcam), GFRA1 (Santa Cruz), PCNA (Santa Cruz), GPR125 (Abcam), and THY1 (Abcam) at a dilution with 1:200 overnight at 4 °C. After washes three times in PBS for 5 min each, the cells were incubated with appropriate FITC-conjugated or rhodamine-conjugated IgG secondary antibodies (Sigma) for 1 hour at room temperature. The nuclei of cells were stained by DAPI (4′ -6-diamidino-2-phenylindole) and the cells were observed the epifluorescence using fluorescence microscope (Leica).

### Western Blots of the Immortalized Human Male Germline Stem Cells

The immortalized human male germline stem cells and human primary SSCs were lysed with RIPA buffer (Santa Cruz) for 30 min on ice. Cell lysates were cleared by centrifugation at 12,000 g, and the concentrations of total proteins were measured by BCA kit (Dingguo Company, China). Twenty micrograms of cell lysates from each sample were resolved by SDS-PAGE (Bio-Rad Laboratories, Richmond, CA), and Western blots were performed according to the protocol as described previously^1^. The chosen antibody included SV40 (Santa Cruz), PCNA (Santa Cruz), RET (Santa Cruz), UCHL1 (AbD Serotec), and ACTB (Proteintech). After extensive washes in PBS, the blots were detected by chemiluminescence (Chemi-Doc XRS, Bio-Rad, Hercules, CA).

### Proliferation Assays of the Immortalized Human Male Germline Stem Cells

Human immortalized male germline stem cells were seeded in 96-well plates at a concentration of 1000 cells/well in 100 μl of DMEM/F12 medium containing 0.5%, 1%, 2%, 5%, 10%, and 15% FBS, and they were incubated at 37 °C in 5% CO_2_ for 7 days. Cellular proliferation was measured every 24 hours according to the protocol of the Cell Counting Kit-8 (CCK-8) assay Kit (DOJINDO). Measurement of the absorbance was performed at 450 nm using a microplate reader, and cell growth curves were drawn to show the proliferation of human SSC line under different concentrations of FBS.

### Xenotransplantation Assays of the Immortalized Human Male Germline Stem Cells

Transplantation assay is the gold standard method for identifying SSC function. The transplantation of immortalized human male germline stem cells was performed according to the procedure as previously described^1^,^38^. Briefly, 20 male ICR mice of 6–8 weeks old were obtained from Shanghai Laboratory Animal Center, Chinese Academy of Sciences, Shanghai, China, and busulfan (40 mg/kg body weight, Sigma, St Louis, MO, USA) was utilized by intraperitoneal injection to deplete male germ cells. Animals were maintained and experiments were carried out strictly in accordance with the care and use of laboratory animals and the related ethical regulation of Ren Ji Hospital, Shanghai Jiao Tong University. Human immortalized germline stem cells were collected and resuspended in DMEM/F12 medium at a concentration of 10^7^/ml. Filtered trypan blue was added to cell suspension and agitated before cell transplantation. Approximately 15 μl of cell suspension were transplanted into the seminiferous tubules of one testis via the efferent duct, while the other testis without cell injection served as negative controls. Eight weeks after cell transplantation, the testes of the recipient mice were collected for preparing the frozen sections and were analyzed through supervision for eGFP expression of human immortalized germline stem cells and immunohistochemistry as described below.

### Immunohistochemistry of the Recipient Mice with Transplantation of the Immortalized Human Male Germline Stem Cells

To assess whether the immortalized human male germline stem cells could survive and proliferate in recipient mouse seminiferous tubules, immunohistochemistry was performed as described previously^18^. Briefly, mouse testes were fixed in 4% PFA, embedded in paraffin, and sectioned at 5 μm thickness. The sections were dewaxed in xylene and rehydrated through a series of graded alcohols from 100% to 50%. Antigen retrieval was performed using the citrate buffer solution for 20 min at 96 °C. To quench endogenous peroxidase activity, sections were treated with 3% hydrogen peroxide. After permeabilization with 0.4% Triton X-100 and blocking with 5% donkey serum (Maibio), the sections were incubated with primary antibodies, including PCNA (Santa Cruz), UCHL1 (AbD Serotec), GPR125 (Abcam), and HumNuc (Abcam) at a 1:100 dilution overnight at 4 °C. After washes three times with PBS, sections were incubated with secondary antibodies to rhodamine-conjugated IgG and/or FITC-conjugated IgG at a 1:200 dilution for 1 hour at room temperature and washed three times with PBS. DAPI was used to stain cell nuclei and the sections were observed the epifluorescence using fluorescence microscope (Nikon Eclipse Ti-S, Nikon Corporation, Tokyo, Japan). To determine the expression of PCNA, MAGEA4 (a kind gift from Professor Giulio C. Spagnoli, University Hospital of Basel, Switzerland), and HumNuc (Abcam), sections were incubated with horse radish peroxidase-conjugated secondary antibody for 1 hour at room temperature and followed by 3, 3-diaminobenzidine (DAB) as a substrate. After immunnostaining, sections were counterstained with hematoxylin and observed under a light microscope (Nikon Eclipse Ti-S, Nikon Corporation, Tokyo, Japan).

### Karyotyping Assays of the Immortalized Human Male Germline Stem Cells

Chromosomal karyotype analysis of the exponentially growing immortalized male germline stem cells at passage 7 was performed pursuant to the procedure described previously^7^,^47^. Briefly, cells were treated with 5 μg/ml colcemid, disrupted in 0.075 M KCl solution, fixed with 3:1 methanol–glacial acetic acid, and dropped onto chilled wet slides. Cells were stained with Giemsa and counted under a microscope. The karyotype was interpreted utilizing the recommendation of the International System for Human Cytogenetic Nomenclature.

### Multiplex PCR Analysis of the Immortalized Human Male Germline Stem Cells

Multiplex PCR was performed to check the expression of numerous Y chromosome genes, including SRY, sY254, sY127, sY86, sY134, sY84 and sY255, in the immortalized human male germline stem cells, according to the procedure as described previously^47^, and PCR without primers served as negative controls.

### Tumor-formation Potential of the Immortalized Human Male Germline Stem Cells by Xenotransplantation Assays

Thirty male nude mice (nu/nu BALB-c) at 5-week-old were used for *in vivo* tumor-formation potential of the immortalized human male germline stem cells. The mice were housed in constant laboratory conditions of a 12-hour light, 12-hour dark cycle and pathogen-free conditions and fed with water and food *ad libitum.* All mice were treated in compliance with the animal care and use guidelines of Ren Ji Hospital animal care and ethics review committee. For xenograft study, the mice were divided into three groups, including control group without cell transplantation, the group transplanted with immortalized human male germline stem cells, and the group transplanted with human prostate cancer cells DU145. In the first group, each nu/nu nude mouse was injected subcutaneously into the left and right flank without cells in a total volume of 100 μl DMEM/F12 and Matrigel (1:1). In the latter two groups, each nu/nu nude mouse was injected subcutaneously into the left and right flank with 10^7^ immortalized human male germline stem cells or 10^7^ human prostate cancer cells DU145 in a total volume of 100 μl DMEM/F12 and Matrigel (1:1). All groups of animals were monitored daily for tumor formation and growth. Two months later, the tissues from mouse transplanted sites were fixed in Bouin’s fixative overnight, embedded in paraffin, and sectioned at 5 μm thickness. The sections were stained with hematoxylin and eosin (H&E) and observed under a microscope (Nikon Eclipse Ti-S, Nikon Corporation, Tokyo, Japan).

### Statistical Analysis

All the values were presented as mean ± SEM from at least three independent experiments. Statistical differences were evaluated using the analysis of variance (ANOVA), and P < 0.05 was considered statistically difference.

## Additional Information

**How to cite this article**: Hou, J. *et al.* Establishment and Characterization of Human Germline Stem Cell Line with Unlimited Proliferation Potentials and no Tumor Formation. *Sci. Rep.* **5**, 16922; doi: 10.1038/srep16922 (2015).

## Figures and Tables

**Table 1:** The primer sequences of genes used for RT-PCR.

Gene	Primer sequence		Product size (bp)	Tm(℃)
*UCHL1*	Forward	CCAATGTCGGGTAGATGA	244	55
	Reverse	CCAATGTCGGGTAGATGA		
*RET*	Forward	CCAATGTCGGGTAGATGA	126	52
	Reverse	CCAATGTCGGGTAGATGA		
*GPR125*	Forward	TACCCTTTGGACTTGGTT	246	49
	Reverse	TACCCTTTGGACTTGGTT		
*GFRA1*	Forward	CCAAAGGGAACAACTGCCTG	410	58
	Reverse	CGGTTGCAGACATCGTTGGA		
*PLZF*	Forward	CGGTTCCTGGATAGTTTGC	317	54
	Reverse	GGGTGGTCGCCTGTATGT		
*MAGEA4*	Forward	CCGAGTCCCTGAAGATG	155	54
	Reverse	CAGGACGATTATCAGAAGG		
*THY1*	Forward	ATCGCTCTCCTGCTAACAGTC	548	51
	Reverse	CTCGTACTGGATGGGTGAACT		
*PCNA*	Forward	CCTGACAAATGCTTGCTGAC	129	55
	Reverse	GCGGGAAGGAGGAAAGTCTA		
*SV40*	Forward	GCTGAGGTGAAGACGGAGAT	200	55
	Reverse	GGGCAACACGGAGTAGATG		
*ACTB*	Forward	CGCACCACTGGCATTGTCAT	200	55
	Reverse	TTCTCCTTGATGTCACGCAC		
