# Corrigendum: Establishment and Characterization of Human Germline Stem Cell Line with Unlimited Proliferation Potentials and no Tumor Formation

**DOI:** 10.1038/srep43195

**Published:** 2017-02-21

**Authors:** Jingmei Hou, Minghui Niu, Linhong Liu, Zijue Zhu, Xiaobo Wang, Min Sun, Qingqing Yuan, Shi Yang, Wenxian Zeng, Yang Liu, Zheng Li, Zuping He

Scientific Reports 5: Article number: 16922; 10.1038/srep16922 published online: 11 20 2015; updated: 02 21 2017.

The export of human derived tissues and related materials from China is currently subject to regulatory restrictions. Consequently, we are unable to share the human cell line described in this paper with researchers outside of China at the present time. However, scientists within China who wish to use this cell line for research only may obtain them from the following company: Cellular Biotech. Inc. www.qzbiotech.com.

